# Treatment of retinopathy of prematurity (ROP)

**Published:** 2018

**Authors:** Renu Puthenvilayil Rajan, Tavisha Udupihille

**Affiliations:** Vitreo Retinal Surgeon: Aravind Eye Hospital, Madurai, Tamil Nadu, India; Consultant Paediatric Ophthalmologist: Sirimavo Bandaranaike Specialised Children's Hospital, Peradeniya, Sri Lanka.

**Figure:**
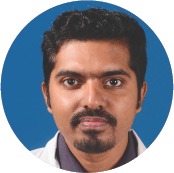
Renu Puthenvilayil Rajan

**Figure:**
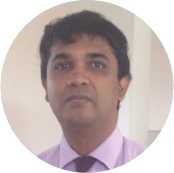
Tavisha Udupihille

**Although laser is the mainstay of ROP treatment, antibodies against vascular endothelial growth factor (VEGF) are being increasingly used. In either case long term follow-up is essential.**

**Figure:**
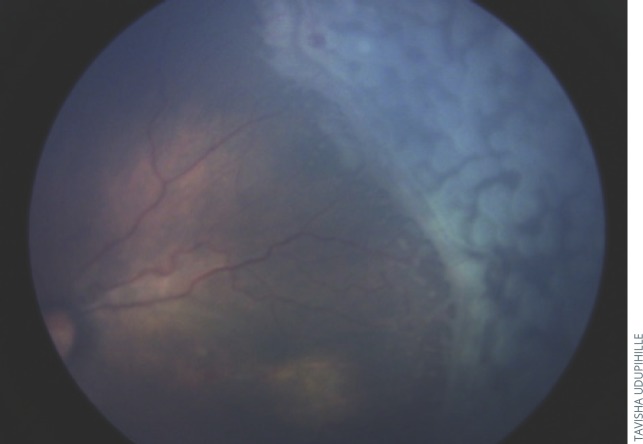
An eye with ROP after laser treatment.

The mainstay of treatment of ROP to date is laser therapy. There is also an increasing use of antibodies against vascular endothelial growth factor (VEGF) either as a primary treatment or as a procedure for selected cases due to failure of primary treatment using laser. It has also been used in situations where laser therapy cannot be instituted due to clinical instability of the baby.

## Which babies require treatment?

Treatment indications are primarily governed by the findings of two clinical trials: Cryo-ROP trial^1^ and Early treatment ROP (ETROP) trial.^2^ The Cryo-ROP trial identified treatment indications are:

“Stage 3 ROP involving a threshold number of at least five contiguous or eight total clock hour sectors of zone 1 or 2, and ‘plus’ disease.”^1^

Although this trial used cryotherapy as the treatment modality its findings have been applied to laser therapy as well. The subsequent ETROP study found benefit in laser treatment of some babies who did not meet the criteria in the Cryo-ROP trial. These indications have been identified in the ETROP study, defined as high risk prethreshold ROP.^2^

- Zone I, any stage ROP with plus disease
- Zone I, stage 3 ROP without plus disease
- Zone II, stage 2 or 3 with plus disease

Zone III, stage 3 with plus disease is not recognised as an indication for treatment in either study, although these babies are sometimes treated. In addition another indication for treatment called Aggressive Posterior ROP (APROP) is increasingly being recognised. This condition is characterised by dilated and tortuous vessels in the posterior pole, although clear definitions and treatment indications are lacking.

## What treatment modalities are available?

Cryotherapy has mainly been superseded by laser therapy. Intra-vitreal injections of VEGF antibodies (bevacizumab and ranibizumab) are also used. Detached retinas that have occurred due to failure of treatment or late presentation require vitreo-retinal surgery.

## Laser treatment

Laser therapy is applied to the region of avascular retina that is anterior to the ridge or demarcation line. Both diode and green (argon or double frequency Neodymium: Yttrium Aluminium Garnet) can be used and delivered via a laser indirect ophthalmoscope. General anaesthesia is ideal for the procedure although sedation and/or topical anaesthesia is used extensively in the South Asian region. The baby has to be monitored during the procedure.

## Anti VEGF therapy

Intra-vitreal injection of bevacizumab or ranibizumab is used in the management of very severe ROP, when laser treatment fails or when the baby is unstable for laser treatment. Bevacizumab eliminates the angiogenic threat of ROP (BEAT-ROP)^3^ and other studies have shown the efficacy of this procedure in causing regression of ROP. However the long term risk to the retina and the baby as a whole have not been studied extensively.

## Follow-up after treatment

Babies need to be followed-up after treatment to ensure that ROP is completely regressed and does not recur. Babies treated with anti-VEGF agents need a longer period of follow-up until the retina is completely vascularised. In addition long term follow-up with regard to the possibility of refractive errors and cortical visual impairment has to be instituted.
